# Mapping between the OBO and OWL ontology languages

**DOI:** 10.1186/2041-1480-2-S1-S3

**Published:** 2011-03-07

**Authors:** Syed Hamid Tirmizi, Stuart Aitken, Dilvan A Moreira, Chris Mungall, Juan Sequeda, Nigam H Shah, Daniel P Miranker

**Affiliations:** 1 Department of Computer Science, The University of Texas at Austin, Austin, Texas 78701, USA; 2 Artificial Intelligence Applications Institute, The University of Edinburgh, Edinburgh EH8 9LE, UK; 3 Informatics Life-Sciences Institute, The University of Edinburgh, Edinburgh EH8 9LE, UK; 4 Department of Computer Science, Mathematics and Computing Institute, University of São Paulo, São Carlos, São Paulo, Brazil; 5 Lawrence Berkeley National Laboratory, Berkeley, California 94720, USA; 6 Center for Biomedical Informatics Research, School of Medicine, Stanford University, Stanford, California 94305, USA; 7 Institute for Cell and Molecular Biology, The University of Texas at Austin, Austin, Texas 78701, USA

## Abstract

**Background:**

Ontologies are commonly used in biomedicine to organize concepts to describe domains such as anatomies, environments, experiment, taxonomies etc. NCBO BioPortal currently hosts about 180 different biomedical ontologies. These ontologies have been mainly expressed in either the Open Biomedical Ontology (OBO) format or the Web Ontology Language (OWL). OBO emerged from the Gene Ontology, and supports most of the biomedical ontology content. In comparison, OWL is a Semantic Web language, and is supported by the World Wide Web consortium together with integral query languages, rule languages and distributed infrastructure for information interchange. These features are highly desirable for the OBO content as well. A convenient method for leveraging these features for OBO ontologies is by transforming OBO ontologies to OWL.

**Results:**

We have developed a methodology for translating OBO ontologies to OWL using the organization of the Semantic Web itself to guide the work. The approach reveals that the constructs of OBO can be grouped together to form a similar layer cake. Thus we were able to decompose the problem into two parts. Most OBO constructs have easy and obvious equivalence to a construct in OWL. A small subset of OBO constructs requires deeper consideration. We have defined transformations for all constructs in an effort to foster a standard *common mapping* between OBO and OWL. Our mapping produces OWL-DL, a Description Logics based subset of OWL with desirable computational properties for efficiency and correctness. Our Java implementation of the mapping is part of the official Gene Ontology project source.

**Conclusions:**

Our transformation system provides a lossless roundtrip mapping for OBO ontologies, i.e. an OBO ontology may be translated to OWL and back without loss of knowledge. In addition, it provides a roadmap for bridging the gap between the two ontology languages in order to enable the use of ontology content in a language independent manner.

## Background

Two ontology based systems, the Open Biomedical Ontologies (OBO) [1] and the Semantic Web [2,3], each associated with a large community are being developed independently. Ontologies in biomedicine are used for organizing biological concepts and representing relationships among them. Major results include the Gene Ontology (GO) [4] and the Zebrafish Anatomy Ontology (ZFA) [5]. OBO format, which originated with GO, continues to evolve in support of the needs of the biomedical community. Over 100 OBO ontologies are available on the NCBO BioPortal [6]. Thus OBO is the backbone for ontology tools in this domain.

**Figure 1:**
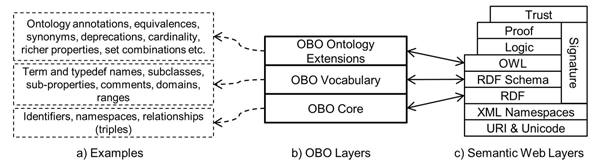
**Layer cakes for OBO and the Semantic Web** A layer cake for OBO, with some examples and a comparison with the Semantic Web layers; the mapping between the two layer cakes is generally quite straightforward, which makes it easy to understand the constructs in OBO and their mappings in OWL.

The Semantic Web is an evolving extension of the World Wide Web based on ontologies. Intended to facilitate search and information integration, and built on the foundations of artificial intelligence, the Semantic Web envisions the Web becoming a global knowledgebase through distributed development of ontologies using formally defined semantics, global identifiers and expressive languages for defining rules and queries on ontologies. The Semantic Web has been organized in the form of a layer cake where each layer provides a representation language of increasing expressive power (see Figure 1). The Web Ontology Language (OWL) [7], a component of the Semantic Web, provides the capability of expressing ontologies in multiple dialects. OWL-DL, a Description Logics based dialect, has become its language of choice due to the availability of reasoning tools. In the biomedical domain, some important ontologies such as NCI Thesaurus [8] and BioPAX [9] have been modelled in OWL.

Given the volume and growth of OBO content, integrating the features promised by Semantic Web technologies with OBO content would provide significant benefit to the biomedical community. One way to provide these features is to create a system that allows back and forth translation of OBO ontologies between the two systems.

This paper describes precisely such a round-trip and the methodology that was followed in the course of its creation. The results in this paper represent a community effort to create a standard transformation mapping, initiated by the OBO foundry. One goal was to reconcile a number of independent efforts. In addition to this paper, a summary of this collaboration is in additional file 1 that lists the transformation choices of the respective contributors and a mediated set of transforms, called the ‘common mapping’. Supplemental material on the mapping is also available [10]. The final results produce OWL-DL, as validated by WonderWeb OWL Ontology Validator [11]. A full implementation was done in Java, and is a part of the Gene Ontology project source [12], hosted at sourceforge.net. It provides a lossless roundtrip mapping for OBO ontologies, i.e. ontologies that are originally in OBO can be translated into OWL and back into OBO.

A basis for reconciling the efforts was an observation that the Semantic Web layer cake itself could serve as a guideline for studying the representation of ontologies in OBO and creating the transformation system. We found that most of OBO can be decomposed into layers with direct correspondence to the Semantic Web layer cake. Compared to an approach that deals with each construct individually, we found that this method gave a better organization to our work and enabled us to identify matches and mismatches between the two languages more efficiently. Discussions became a two step process where it was first determined if an OBO construct had a clear correspondence to a Semantic Web layer, with respect to its intended expressive power, and if so, to which level it belonged. It followed that constructs that fell into the same equivalence class should be handled similarly. Deep discussion could be limited to those OBO constructs that could not be easily situated in this structure. These include, (1) local identifiers in OBO compared to global identifiers in OWL, (2) various kinds of synonym elements in OBO, and (3) defining subsets of OBO ontology. Even these constructs can be expressed in OWL-DL, albeit not by obvious construct substitution. We conclude that OWL-DL is strictly more expressive than OBO.

An additional consequence of this work is that, in effect, it defines a subset of OWL-DL that captures the expressive power of OBO and can be seen as a way of introducing formal semantics to OBO. We include a discussion of how OWL tools can be restricted to this subset so as to assure that ontologies developed with OWL tools may be translated to OBO.  Similarly and perhaps more importantly, how to assure that OWL tools do not break OBO ontologies that have been translated to OWL such that, after using OWL tools, an updated ontology may be returned to OBO form. The exception handling in the Java based OWL to OBO translator was developed such that the translator itself serves double duty as a validator for this subset of OWL. At least two biomedical ontology tools, OBO-Edit [13] and Morphster [14] already exploit this translator.

OWL and OBO continue to evolve. OWL 2 [15] has recently been ratified by the World Wide Web Consortium, and a new version of OBO (1.3) is under active development [16]. Given that the older versions of these languages still support most ontologies, we have focused on those versions. However, later in the paper we provide a discussion on the new versions and their impact on the transformation system.

### Related work

Each of the authors of this paper, as well as Mikel Egana, Erick Antezana, and LexBio group at Mayo Clinic, contributed some earlier independent effort at creating a transformation system [17-19]. The results of these efforts are documented in our spreadsheet as well. No single effort survived in its entirety in the common mapping.

Another independent and important effort was that of Golbreich et al [20,21] (hereafter Golbreich) that was not included in the standardized mappings. Golbreich developed a BNF grammar for OBO syntax, as well as a mapping between OBO and OWL 1.1 (now known as OWL 2). The differences between the Golbreich work and the common mapping effort presented in this paper comprise a difference of methodology and practical focus. Golbreich’s work laid out valuable syntactic groundwork to formalize the semantics of a large subset of OBO. Much like most of the other first efforts, a complete transformation system was not specified. This particular effort deferred resolving OBO annotations, synonyms, subsets, and deprecation tags. Golbreich’s work also did not address the mapping of local identifiers in OBO into global identifiers. However, the transformations that are specified by Golbreich are largely consistent with the common mappings.

### Definitions

#### Ontology

In knowledge-based systems, an ontology is a vocabulary of concepts and describable relationships among them [22]. Ontologies are extensively used in areas like artificial intelligence [23,24], the Semantic Web [7,25-27] and biology [4-6] as a form of knowledge representation. They generally describe individual objects (or instances), classes of objects, attributes, relationship types, and relationships among classes and objects within a domain.

#### OBO ontologies

An ontology in OBO consists of two parts; the first part is the header that contains tag-value pairs describing the ontology, and the other part contains the domain knowledge described using term and typedef (more commonly known as a relationship type) stanzas [28]. A stanza generally defines a concept (term or typedef) and contains a set of tag-value pairs to describe it. To the terms and typedefs defined in OBO ontologies are assigned local IDs and namespaces.

The OBO format is human friendly, and useful GUI-based tools like OBO-Edit are available for building ontologies in it [13]. We deal with OBO version 1.2, and refer to it as simply OBO in this paper.

#### Semantic Web ontologies

The Semantic Web ontologies give well-defined meaning to the content on the World Wide Web and enables computers and people to work in cooperation. Some key technologies that form the Semantic Web are:

1. Resource Description Framework (RDF) [29] can express meaning of data using triples. A triple is a binary predicate that defines a relationship between two entities.

2. The Semantic Web uses Universal Resource Identifiers (URIs). This means that each entity gets a globally unique identifier.

3. RDF Schema (RDF-S) and Web Ontology Language (OWL) are ontology languages. RDF-S allows description of valid classes and relationship types for an application, and some properties like subclasses, domains, ranges etc. OWL further allows describing constraints on instances and provides both ontology level and concept level annotations, set combinations, equivalences, cardinalities, deprecated content etc.

A common syntax for representing ontologies on the Semantic Web is RDF/XML. OWL is based on RDF and RDF-S, and on occasion, we use OWL as an encompassing term for all these languages.

## Results

### OBO and the Semantic Web layers

The Semantic Web was envisioned as an expressive hierarchy that is often illustrated as a layer cake [3] (see Figure 1c). At the beginning of this research it was our conjecture that the precise organization of the hierarchy transcends the Semantic Web and could be used, retroactively, to formalize the structure of other data and concept modelling systems. Thus, as a first step towards the creation of a transformation mechanism between OBO and OWL, we created a layer cake for OBO whose structure mirrored that of the Semantic Web layer cake. This allowed us to identify straightforward mappings as well as the cases that do not match as well. We term this the ‘two layer cakes’ methodology. This methodology has also been successfully applied towards the transformation of SQL databases into OWL ontologies [30].

#### OBO layer cake

We methodically examined each of the constructs of OBO. We find that most of OBO can be decomposed into layers with direct correspondence to the Semantic Web: OBO Core, OBO Vocabulary, and OBO Ontology Extensions (see Figure 1a, 1b).

1. **OBO Core:** In OBO, a concept can either be a term (class) or a typedef (relationship type). OBO Core deals with assigning IDs and ID spaces to concepts, and representing relationships as triples.

2. **OBO Vocabulary:** OBO Vocabulary allows annotating concepts with metadata like names and comments. It also supports describing sub-class and sub-property relationship types, as well as the domains and ranges of typedefs.

3. **OBO Ontology Extensions:** In addition to concept-level tags, OBO Ontology Extensions (OBO-OE) layer defines tags for expressing metadata on the entire ontology as well. It also allows defining synonyms, equivalences and deprecation of OBO concepts. OBO-OE layer can also express specific properties of OBO terms (e.g. set combinations, disjoints etc.), and typedefs (e.g. transitivity, uniqueness, symmetry, cardinalities).

Table 1 provides assignments of OBO constructs to appropriate layers in the OBO layer cake.

**Table 1:** Layer cake assignments for OBO constructs

**OBO Core:** id, idspace, relationship
**OBO Vocabulary:** name, definition, comment, is_a, domain, range

**OBO Ontology Extensions:** format-version, version, date, saved-by, auto-generated-by, namespace, default-namespace, subsetdef, alt_id, relationship, subset, synonym, is_obsolete, is_cyclic, is_transitive, is_symmetric, import, synonymtypedef, intersection_of, union_of, disjoint_from, replaced_by, consider, inverse_of, transitive_over

Since we mostly have an exact mapping of layers between the two languages (see Figure 1), deciding which constructs to use for each kind of transformation is simplified. OBO Core tags can be transformed using RDF. OBO Vocabulary tags require using RDF Schema constructs. OBO Ontology Extensions tags require constructs defined in OWL. 

#### Incompatibilities between OBO and OWL

We classify incompatibilities between the two languages into one of the two categories. First, in certain cases, the semantic equivalent of a construct in one language is missing from the other language. Second, sometimes the semantics of constructs in OBO are not sufficiently well-defined to map to a formally defined OWL construct, which forces us to define new vocabulary in OWL in order to allow the lossless transformation.

1. Entities in OWL have globally unique identifiers (URIs). On the other hand, OBO allows local identifiers. Transforming OBO into OWL requires transforming the local identifiers in an OBO ontology into URIs. Also, in order to make the roundtrip possible, it is necessary to extract the local identifier back from the URI.

2. OBO language has the ‘subset’ construct, which does not have an equivalent construct in OWL. An OBO subset is a collection of terms only, and is defined as a part of an ontology. An ontology can contain multiple subsets and each term can be a part of multiple subsets. In order to make the transformation possible, we need to define an OWL construct equivalent to OBO subset, and some relationship concepts to represent terms being in a subset, and a subset being a part of an ontology.

3. There are multiple kinds of synonym tags in OBO, e.g. related, narrow, broad, exact etc. The differences between these constructs are not formally documented. This requires defining new concepts in OWL, which can perhaps be mapped to new or already existing constructs in OWL.

Elements of OBO “missing” in Semantic Web are few, and can still be constructed in OWL. Thus, OBO ontologies may be translated to Semantic Web. However, in order to make the roundtrip possible, we find it important to store some ancillary information about the OBO ontology in the OWL file, e.g. a base URI etc., so it can be translated back without any loss of knowledge. It is important to note that even changing a local identifier within the whole knowledgebase is counted as loss of knowledge from the original source, even if the overall structure of the ontology remains intact.

The presence of such incompatibilities requires us to make some complex choices regarding the transformation process. Our solutions to these problems are explained in detail later.

#### OBO and sub-languages of OWL

OWL has three increasingly expressive sublanguages; OWL Lite, OWL DL and OWL Full. Each of these sublanguages extends its simpler predecessor with richer constructs that affect the computational completeness and decidability of the ontology.

Our investigation shows that a major portion of the OBO Ontology Extensions maps to OWL Lite and provides similar level of expressiveness. Overall, OBO features are a strict subset of OWL DL.

In OBO, the definition of a term, or a typedef, is rigid and not as expressive as OWL Full. OWL Full allows restrictions to be applied on the language elements themselves [7,26]. In other words, an OWL Full Class can also be an OWL Full Property and an Instance and vice versa. Such features are not supported in OBO.

Recall, the primary concern is the use of the Semantic Web technology and tools for OBO ontologies. Thus, that OBO is less expressive than OWL is the convenient direction of containment. It does mean that round trips cannot be supported unless the editing of any OBO ontology while in their OWL representation is restricted. We talk about editing of transformed ontologies while in OWL language in a later section.

While transforming OBO ontologies into OWL, we must ensure producing a representation that can be used by description logic based inference engines. One of the intended goals of our transformation is to produce OWL DL, and not OWL Full.

### Transformation metadata and rules

In this section, we present some of the rules for the transformation of OBO ontologies into OWL. For more complex transformations we describe the transformations and explain our approach.

In order to facilitate the transformation, we have defined a set of OWL meta-classes that correspond to the vocabulary of OBO tags. Complete listing of mappings between OBO and OWL are available in additional file 1.

#### Simple transformation rules

Most of the transformations follow simple rules. For most header and term/typedef tags, there is a one-to-one correspondence between OBO tags and OWL elements, either pre-existing or newly defined. In this section, we list the elements with this kind of simple transformation. Table 2 Example A provides some examples.

**Table 2:** OBO examples and corresponding OWL mappings

OBO	OWL
[Typedef] id: part_of name: part of is_transitive: true	<owl:TransitiveProperty rdf:about="…#part_of"> <rdfs:label>part of</rdfs:label> </owl:TransitiveProperty>

*Example A Simple transformations: name, transitivity*	

[Term] id: ZFA:0000434 name: skeletal system is_a: ZFA:0001439	<owl:Class rdf:about="...#ZFA_0000434"> <rdfs:label>skeletal system</rdfs:label> <rdfs:subClassOf rdf:resource="...#ZFA_0001439"/> </owl:Class>

*Example B Transformation of ‘is-a’*	

[Term] id: ZFA:0001439 name: anatomical system relationship: part_of ZFA:0001094	<owl:Class rdf:about= “…#ZFA_0001439”> <rdfs:label>anatomical system</rdfs:label> <rdfs:subClassOf><owl:Restriction> <owl:onProperty rdf:resource = “…#part_of” /> <owl:someValuesFrom rdf:resource = “…#ZFA_0001094” /> </owl:Restriction></rdfs:subClassOf> </owl:Class>

*Example C Transformation of a relationship*	

[Term] id: ZFA:0000437 name: stomach is_obsolete: true	<owl:Class rdf:about="&oboInOwl;ObsoleteClass"/> <owl:Class rdf:about="...#ZFA_0000437"> <rdfs:label>stomach</rdfs:label> <rdfs:subClassOf rdf:resource="&oboInOwl;ObsoleteClass"/> </owl:Class>

*Example D Transformation of obsolete term*	

OBO examples in this table have been taken from ZFA.

**Header:** The set of tag-value pairs at the start of an OBO file, before the definition of the first term or typedef, is the header of the ontology.

When translated into OWL language, each of the OBO header tags gets translated into the corresponding OWL element. The whole ontology header is contained in the *owl:Ontology* element in the new OWL file, and can appear anywhere within the file, as opposed to the start of file in OBO language.

**Terms:** A term in OBO is a class in OWL. So, a term declaration is translated into an *owl:Class* element and the tags associated with a term are contained within this element. Some tags that have straightforward transformations to OWL elements are:

1. The elements for ‘name’ and ‘comment’ about a term fall into the OBO Vocabulary layer, and are translated into *rdfs:label* and *rdfs:comment* respectively. A ‘definition’ tag is translated into *hasDefinition* annotation property, and is therefore placed in the OBO Ontology Extensions layer.

2. The ‘is_a’ tag in OBO specifies a subclass relationship, and is placed in the OBO Vocabulary layer. It is translated into an *rdfs:subClassOf* element (Table 2 Example B).

**Typedefs:** A typedef in OBO is an object property in OWL. A typedef stanza in an OBO file is translated into an *owl:ObjectProperty* element in OWL. The other information associated with the typedef is expressed as elements nested within this element. Some simple transformations are:

1. OBO typedefs can have associated domains and ranges. These are expressed by ‘domain’ and ‘range’ tags, and are in the OBO Vocabulary layer. These tags are translated into RDF Schema defined elements *rdfs:domain* and *rdfs:range* respectively.

2. Just like subclasses for terms, a property can be a sub-property to another property. A sub-property relationship is expressed using the ‘is_a’ tag, from OBO Vocabulary layer, in a typedef stanza. This tag is translated into an *rdfs:subPropertyOf* element defined in RDF Schema. 

3. Typedefs may be cyclic (‘is_cyclic’ tag), transitive (‘is_transitive’ tag) or symmetric (‘is_symmetric’ tag). These tags fall into the OBO Ontology Extensions layer. The corresponding elements in OWL are annotation property *isCyclic*, and property types *owl:TransitiveProperty* and *owl:SymmetricProperty* respectively. The *isCyclic* property specifies a Boolean value.

#### Identifiers and ID spaces

OBO has a local identifier scheme. As OBO evolves, ID spaces have been introduced to allow specifying global identifiers. OBO identifiers have no defined syntax, but they are recommended to be of the form: *“<IDSPACE>:<LOCALID>”*

However, OBO ontologies may contain flat identifiers, ones that do not mention the ID space. OBO identifiers must be converted to URIs for use in OWL. The rules for converting OBO identifiers to URIs in the current mapping are as follows:

If the OBO header declares an ID space of the form: *“idspace: GO http://www.go.org/owl#”,* all OBO identifiers with the prefix *GO:* will be mapped to the provided URI, e.g. *“http://www.go.org/owl#GO_0000001”*.

If an OBO ID space prefix does not have a declaration in the header, all identifiers that mention that prefix will be transformed using a default base URI, for example an identifier of the form *“SO:0000001”* will become *“<default-base-uri>SO_0000001”*. In case the OBO identifier is flat, e.g. *foo*, the transformation again uses the default base URI to create *“<default-base-uri>UNDEFINED_foo”*. Notice that the URI contains *“UNDEFINED_”*, which clarifies that the URI should be translated into a flat identifier when translating the OWL version back to OBO. Flat identifiers are discouraged in OBO since they are not globally unique. Our transformation scheme only attempts to enable the roundtrip, and does not guarantee uniqueness of the identifiers.

Typedefs defined in OBO Relations Ontology [31] are often used as a common vocabulary in OBO ontologies. Such typedefs have OBO identifiers prefixed with ID space *OBO_REL*. OBO ontologies assume the presence of this ID space with URI *“http://www.obofoundry.org/ro/ro.owl”* even if it is not explicitly stated. When translated into OWL, an XML namespace *xmlns:oboRel* with the same URI is added to the ontology, and the newly created object property is assigned that namespace. As a result, we ensure that all Relations Ontology constructs are mapped to the same URIs across ontologies.

#### Relationships

Relationships between OBO terms can be defined using the ‘relationship’ tag. A defined relationship is like a binary predicate and consists of a subject (the term being described in the stanza), a relationship type and an object.

There are multiple kinds of restrictions on relationships that can be expressed using OWL. OBO specifications do not specify any formal semantics for the ‘relationship’ tag that match a specific relationship type restriction defined in OWL. Therefore, we have selected the most general restriction to transform OBO relationships into OWL.

An example of relationship transformation is shown in Table 2 Example C. The *owl:someValuesFrom* element specifies the type of restriction that is applied to the OWL relationship. This restriction is similar to the existential quantifier of predicate logic [7,26]. In the existing OBO ontology content, we have only seen OBO relationships of this kind. It is possible that some ontologies use a different semantics of relationships. Currently, we do not have a way of differentiating between the two uses of OBO relationships so our transformation is based on the common semantics.

#### Subsets

Terms in an OBO ontology can be organized into subsets. A term can belong to multiple subsets.

In order to declare a subset, a value for the tag ‘subsetdef’ is specified in the OBO ontology header. This value consists of a subset ID (or subset name) and a quoted description about the subset. A term can be assigned to a defined subset using the ‘subset’ tag. Multiple ‘subset’ tags are used to assign the term to multiple subsets of the ontology.

When the ontology is translated into OWL, the mapping of subsets is one of the more complex processes. This is due to the fact that subsets do not have a semantic equivalent in OWL. Therefore, we use some OWL features to construct elements that serve as subsets. Subsets fall in the OBO Ontology Extensions in the OBO layer cake.

The local ID (or name) assigned to the subset, which is locally unique, becomes the OWL ID of a subset resource. A subset resource is declared using an *oboInOwl:Subset* element. The *inSubset* annotation is used to assign terms to a subset, and it is expressed within the *owl:Class* element.

#### Obsolete content

OBO format supports obsolete content. A term or typedef can be marked as obsolete using the ‘is_obsolete’ tag with a ‘true’ Boolean value. The ‘is_obsolete’ tag is in the OBO Ontology Extensions.

Obsolete terms and typedefs are not allowed to have any relationships with other terms or typedefs, including the subclass and sub-property relationships.

When translated into OWL, an obsolete term becomes a subclass of *oboInOwl:ObsoleteClass* (Table 2 Example D). Similarly, an obsolete typedef becomes a subproperty of *oboInOwl:ObsoleteProperty*.

Notice that while OWL provides elements to handle deprecation, obsolescence in OBO has different semantics, hence requires a different mapping.

## Discussion

### OBO semantics by transformation

The transformation system has the additional effect of formalizing the semantics of the OBO language. The semantics of OBO are operationally defined by means of GO and the software systems that support GO. The semantics of OWL have been formally defined using model theory [25,29]. Though we have not written it out, a formal document specifying (or suggesting) OBO semantics can be generated. The contents of that document would comprise an enumeration of the pair-wise mapping of constructs between the two languages, restating, in each mapping, the semantics stated for the involved OWL construct.

In Table 3, we present a few examples where our transformation mapping could provide formal semantics for OBO constructs, taken directly from OWL semantics specifications. So,

1. *x is_a y*: all instances of *x* are also instances of *y*.

2. *x is domain of y*: the subject entity for all relationships of type *y* is an instance of *x*.

3. *x is disjoint from y*: *x* and *y* do not have any common instances.

**Table 3:** Semantics for OBO using OWL mappings

Description	OBO	OWL	Semantics
x is a subclass of y	is_a	rdfs:subClassOf	CEXT(x) ⊆ CEXT(y)
x is a sub-property of y	is_a	rdfs:subPropertyOf	EXT(x) ⊆ EXT(y)
x is the domain of property y	domain	rdfs:domain	<z,w> ∈ EXT(y) impliesz ∈ CEXT(x)
x is the range of property y	range	rdfs:range	<w,z> ∈ EXT(y) impliesz ∈ CEXT(x)
x is disjoint from y	disjoint_from	owl:disjointWith	CEXT(x) ∩ CEXT(y) = {}
p is a transitive property	is_transitive	owl:TransitiveProperty	<x,y>,<y,z> ∈ EXT(p) implies <x,z> ∈ EXT(p)

CEXT(c): the set of instances of class c; EXT(p): the set of pairs <x,y> related by property p.

While the identification is straightforward in these cases, in certain other situations, it is not very clear. Finding the semantics of relationships in OBO is one such case. OBO specifications do not provide the semantics of the construct used to specify relationships between two terms using a typedef. Therefore, it is hard to decide which of the available relationship constraints in OWL (*owl:allValuesFrom*, *owl:someValuesFrom*) to use, the former being similar to a universal quantifier, and the latter to an existential quantifier. In our transformations, we use *owl:someValuesFrom*, since already built ontologies show examples of use of OBO relationship construct in a way compatible to that of *owl:someValuesFrom*. We recommend that in practice the semantics of OBO relationships always match the *owl:someValuesFrom* restriction.

Other OBO tags that do not clearly match with OWL elements, such as synonyms and subsets, as well as the semantics for the ‘is_obsolete’ tag also present a more significant challenge in the identification of semantics.

### Updating OBO ontologies in OWL

The set of constructs for ontology representation provided by OWL is considerably larger than the set of constructs provided by OBO. Therefore, in order to allow roundtrip transformations on OBO ontologies, it is important to restrict the editing of such ontologies per some guidelines while they are being represented in OWL.

Our transformation mappings essentially provide a subset of OWL elements that may be used for adding or updating contents of the ontology.

Compared to the general use of OWL, there are two key points to keep in mind:

1. To create relationships, use *owl:someValuesFrom* relations, since OBO does not have a corresponding relationship mechanism for *owl:alValuesFrom*.

2. Obsolescence of terms in the ontology should be done using the obsolete elements *oboInOwl:ObsoleteClass* and *oboInOwl:ObsoleteProperty*. OWL has seemingly similar, but semantically different deprecation elements, which must not be used for obsolescence.

### Interconnecting OBO and the Semantic Web

The implications of our work in providing semantics to OBO strongly suggest the use of this mapping as a potential bridge between the OBO and the Semantic Web worlds. Compared to the existing work by Golbreich et al. [20,21], our ability to make roundtrips between OBO and OWL could enable seamless interconnections between the two worlds. Our roundtrip tool could also be used as a validator for ontologies updated in OWL.

It is common for biologists to develop and refine their OBO ontologies as their work progresses. Our work provides a path for accessing and querying the Semantic Web as well as OBO content in an integrated fashion, and to assimilate linked data available on the Semantic Web.

An implementation of our roundtrip mappings is provided by the Morphster tool [14] to jumpstart the integration of OBO ontologies with the Semantic Web. Morphster has successfully accomplished the use of a Semantic Web based triple store Jena SDB [32] for storage of large OBO ontologies and querying by the SPARQL query language for RDF. It also enables the use of XML Web Services with OBO ontologies to obtain and link diverse data such as images from Morphbank [33], and authoritative taxonomic names from uBio [34] etc.

### OBO 1.3 and OWL 2

OBO and OWL both continue to evolve as ontology languages, providing new features based on real applications and user experience. A new version of OWL, commonly known as OWL 2 [15], has recently been ratified by the World Wide Web Consortium. Meanwhile, a new version of OBO, OBO 1.3, is under active development with draft documents available for comment [16]. As the languages change, tools as well as ontology content will be updated to utilize their new features.

Of particular concern to our work are *the changes that are taking place in each language* and *their impact on the transformations*. In this section, we discuss our understanding of these issues.

New features of OWL 2 mainly concern easier syntax for common ontology statements and new constructs that increase expressivity. Hence, we can expect simpler transformation rules for going from OBO to OWL 2.

The biomedical ontology community now understands that OBO and OWL are both useful ontology languages and the intention is to make these languages entirely inter-convertible in the long term. One of the objectives behind the updates to OBO is to bring the feature set of OBO 1.3 closer to that of OWL.

● OBO 1.3 promises to provide a specification of formal syntax and semantics, hence taking a big step towards making provably correct mappings to OWL possible. The syntax for OBO 1.3 is specified as a BNF grammar, and the semantics are defined using the Obolog language, a collection of logical constructs defined using the ISO standard Common Logic [35]. In addition to the logical semantics of Obolog, the new specifications will also provide interpretations for Obolog to simplify translations into OWL-DL as well as OWL 2.

**Figure 2:**
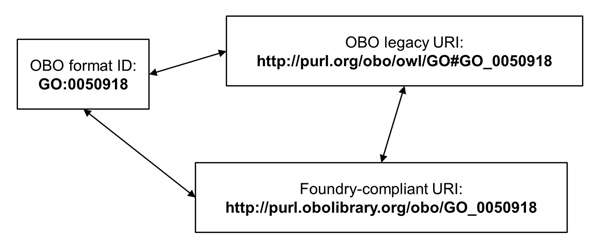
**Mappings between OBO Ids and URIs** A mapping between the existing OBO Ids, newly recommended Foundry-compliant URIs, and the URIs produced by the standard mapping, mentioned as OBO legacy URI. This figure has been taken from the draft of the recommendation, and refers to the mappings of Ids described in the recommendation document.

● The new version of OBO will accompany a recommendation [36] for globally unique identifiers for OBO that will have a one to one mapping with OBO Foundry compliant URIs, hence making the ID mapping obvious. The design goals behind this recommendation are to make sure that the URIs resolve to useful information about an OBO term, and that it is possible to maintain those URIs over time so they keep pointing to useful information. The recommendation document provides an example of how existing OBO IDs, new URIs, and existing transformed URI from the standard mapping may be related in the future (see Figure 2).

● The new version of OBO will introduce new supported stanzas (sections) of OBO ontologies, i.e., ‘Annotation’ and ‘Formula’. Annotation stanzas will allow the representation of annotations, and to attach metadata to them. Formula stanzas will be used to represent logical or mathematical formulas. A transformation system for OBO 1.3 will need to accommodate these stanzas as well.

## Conclusions

Building ontologies is not a new idea for the biology community, and precedes the development of the Semantic Web. While ontologies are a central part of the architecture of the Semantic Web, the Semantic Web vision includes a broad range of technologies from the Artificial Intelligence field, such as inferring and querying mechanisms, as well as additional elements for distributed computing, such as global identifiers and the use of XML and HTTP as middleware. OBO, on the other hand, has appropriate tool support for building ontologies and hosts a number of important biomedical ontologies. Hence the OBO community has the biggest and most immediate need for the features being developed by the Semantic Web community.

We have standardized the mapping between the two systems to allow the OBO community to utilize the tool base developed for the Semantic Web world. We have indirectly formalized the semantics of OBO by creating a roundtrip transformation between OBO and OWL. We have also implemented our transformation tool in Java and it is available as part of the open source Gene Ontology project, and also as a web service. We believe our work is an important step towards building interoperable knowledge bases between OBO and the Semantic Web communities.

A key difference between the OBO community and the Semantic Web is the methodology for content development across ontologies. The Semantic Web has adapted a completely distributed development mechanism for ontologies that may be integrated using URIs. On the other hand, the OBO community uses a hybrid of centralized and distributed development. While the users of OBO develop ontologies independently, the OBO foundry has the goal of collaboratively creating a suite of orthogonal interoperable reference ontologies, such as the Relations Ontology, in the biomedical domain. Our transformation system enriches the Semantic Web by providing this additional structured ontology content and the access to the wealth of data annotated using it.

## Epilogue

As of September 2010, OBO 1.3 has been deprecated, to be replaced by OBO 1.4. In addition to describing the syntax and semantics of OBO 1.4, work is in progress on defining a mapping for the new version of OBO to OWL2-DL. The new mappings are expected to be a part of the final specification of OBO 1.4. Readers should refer to the editor's draft [37] for further developments.

## Methods

Based on the mapping rules, we have implemented a Java implementation of the OBO to OWL transformation. Our implementation is part of the official Gene Ontology project source [12]. Gene Ontology project is an open source project on Sourceforge.net, and is home to the OBO ontology editor OBO-Edit. Our implementation is part of the OBO API that provides data structures for storing OBO ontologies, as well as read and write capabilities for OBO and OWL, among other operations. The source code for our transformation tool is available at [38].

Finally, we have deployed our transformation as a web service for general use: *http://www.cs.utexas.edu/~hamid/oboowl.html*

In the OBO API, we have created *NCBOOboInOWLMetadataMapping* class in the package *org.obo.owl.datamodel.impl*. This class implements the roundtrip mapping between OBO and OWL. In order to provide console-based use of the transformation tool, we have created *Obo2Owl* and *Owl2Obo* classes in *org.obo.owl.test* package.

In order to evaluate the OWL output of our implementation, we have tested our tool on Gene Ontology, Zebrafish Anatomical Ontology, Spider Ontology and Adult Mouse Gross Anatomy, obtained from NCBO BioPortal. After transformation of these ontologies into OWL, we have successfully loaded the OWL files into Protégé [39], an ontology development tool for the Semantic Web. Using the ‘summary’ feature of Protégé, we have compared the overall class and object property count with the term and typedef count obtained for the original OBO file, using OBO-Edit’s ‘extended information’ feature. The results of the comparison (Table 4) show equal values for both versions of the ontologies. Similarly, for testing the roundtrip, we compared the original OBO file with the roundtrip version, again using OBO-Edit’s ‘extended information’ feature. Our evaluation showed that the two OBO ontologies had the same term and typedef counts (Table 4).

**Table 4:** Evaluation results from the roundtrip transformations

Ontology	Original OBO	OWL Translation	Roundtrip OBO
ZFA	Terms: 2219 Typedefs: 4	Classes: 2219 Object Properties: 4	Terms: 2219 Typedefs: 4

MA	Terms: 2882 Typedefs: 1	Classes: 2882 Object Properties: 1	Terms: 2882 Typedefs: 1

SPD	Terms: 494 Typedefs: 1	Classes: 494 Object Properties: 1	Terms: 494 Typedefs: 1

GO	Terms: 28667 Typedefs: 5	Classes: 28667 Object Properties: 5	Classes: 28667 Typedefs: 5

Class counts do not include obsolete classes, or ancillary information required for roundtrips. ZFA = Zebrafish Anatomical Ontology, MA = Adult Mouse Gross Anatomy, SPD = Spider Ontology, GO = Gene Ontology.

## List of abbreviations used

BNF: Backus-Naur Form; GO: Gene Ontology; GUI: Graphical User Interface; HTTP: Hypertext Transfer Protocol; NCBO: The National Center for Biomedical Ontology; OBO: Open Biomedical Ontology; OWL: Web Ontology Language; OWL-DL: OWL Description Logics subset; RDF: Resource Description Framework; RDF-S: RDF Schema; SPARQL: SPARQL Protocol and RDF Query Language; SQL: Stuctured Query Language; URI: Universal Resource Identifier; XML: Extensible Markup Language; ZFA: Zebrafish Anatomy

## Competing interests

The authors declare that they have no competing interests.

## Authors' contributions

All the authors provided their early transformation systems and worked towards the reconciliation of the efforts to create the standard mapping. SHT drafted the manuscript, implemented the Java version, ran the tests and deployed the web service. DAM developed the original roundtrip software for Protégé and OBO import/export plug-ins for Protégé and OBO-Edit. All authors critically reviewed the manuscript, and approved the final version.

## Supplementary Material

Additional file 1 Description: Summary of final mappings, and original independent mappings Click here for file
